# Metabolomics-Driven Nutraceutical Evaluation of Diverse Green Tea Cultivars

**DOI:** 10.1371/journal.pone.0023426

**Published:** 2011-08-10

**Authors:** Yoshinori Fujimura, Kana Kurihara, Megumi Ida, Reia Kosaka, Daisuke Miura, Hiroyuki Wariishi, Mari Maeda-Yamamoto, Atsushi Nesumi, Takeshi Saito, Tomomasa Kanda, Koji Yamada, Hirofumi Tachibana

**Affiliations:** 1 Innovation Center for Medical Redox Navigation, Kyushu University, Higashi-ku, Fukuoka, Japan; 2 Faculty of Agriculture, Kyushu University, Higashi-ku, Fukuoka, Japan; 3 Bio-Architecture Center, Kyushu University, Higashi-ku, Fukuoka, Japan; 4 National Institute of Vegetable and Tea Sciences, National Agriculture and Food Research Organization, Shimada, Shizuoka, Japan; 5 Asahi Breweries Ltd., Moriya, Ibaraki, Japan; East Carolina University, United States of America

## Abstract

**Background:**

Green tea has various health promotion effects. Although there are numerous tea cultivars, little is known about the differences in their nutraceutical properties. Metabolic profiling techniques can provide information on the relationship between the metabolome and factors such as phenotype or quality. Here, we performed metabolomic analyses to explore the relationship between the metabolome and health-promoting attributes (bioactivity) of diverse Japanese green tea cultivars.

**Methodology/Principal Findings:**

We investigated the ability of leaf extracts from 43 Japanese green tea cultivars to inhibit thrombin-induced phosphorylation of myosin regulatory light chain (MRLC) in human umbilical vein endothelial cells (HUVECs). This thrombin-induced phosphorylation is a potential hallmark of vascular endothelial dysfunction. Among the tested cultivars, Cha Chuukanbohon Nou-6 (Nou-6) and Sunrouge (SR) strongly inhibited MRLC phosphorylation. To evaluate the bioactivity of green tea cultivars using a metabolomics approach, the metabolite profiles of all tea extracts were determined by high-performance liquid chromatography-mass spectrometry (LC-MS). Multivariate statistical analyses, principal component analysis (PCA) and orthogonal partial least-squares-discriminant analysis (OPLS-DA), revealed differences among green tea cultivars with respect to their ability to inhibit MRLC phosphorylation. In the SR cultivar, polyphenols were associated with its unique metabolic profile and its bioactivity. In addition, using partial least-squares (PLS) regression analysis, we succeeded in constructing a reliable bioactivity-prediction model to predict the inhibitory effect of tea cultivars based on their metabolome. This model was based on certain identified metabolites that were associated with bioactivity. When added to an extract from the non-bioactive cultivar Yabukita, several metabolites enriched in SR were able to transform the extract into a bioactive extract.

**Conclusions/Significance:**

Our findings suggest that metabolic profiling is a useful approach for nutraceutical evaluation of the health promotion effects of diverse tea cultivars. This may propose a novel strategy for functional food design.

## Introduction

Natural products derived from medicinal plants are an abundant source of biologically active compounds, many of which have formed the basis for development of nutraceuticals and pharmaceuticals [1]. Tea (*Camellia sinensis* L.) is a popular beverage worldwide, and because of its possible health effects, it has received considerable attention as a medicinal herb [2]. There are three main types of tea, which differ according to the fermentation process; green (unfermented), oolong (semi-fermented), and black (fermented). Green tea constituents show various biological and pharmacological activities, such as anti-carcinogenic, anti-metastatic, anti-oxidative, anti-hypertensive, and anti-hypercholesterolemic activities [2]–[5]. The chemical components of tea vary according to species/cultivar, environment, growth, storage conditions, and leaf quality [6]. In most cases, the quality and bioactive functions (i.e., the health promotion effects in human and animal models) of tea are defined by their specific compositions.

The functional biochemistry of plants is very diverse. The concentrations of many compounds vary widely, and metabolomic analyses are required to determine all metabolites in plant extracts. Among many analytical platforms, mass spectrometry (MS) is the most sensitive and selective technique, and thus it is the method of choice for metabolomic research on plants [7]. LC-MS can be adapted to a wide range of molecules, such as secondary metabolites [8]. Metabolomic studies coupled with chemometric methods including principal component analysis (PCA) and partial least-squares (PLS) regression analysis have been used to explore the relationships between the metabolome of diverse plant species and their genotype, origin, vintage, quality, or other specific attributes [9]–[12]. Metabolic profiling techniques are often used to evaluate the nutraceutical (nutritional or physiological) value of a single plant cultivar for quality control and breading. In the field of nutraceutical (functional food) research, such techniques have been used to identify subtle metabolic differences among individuals or among different environmental conditions, e.g., diet [1], [13]. However, to date, there has been little research on the use of metabolic profiling to compare or predict the nutraceutical (bioactive) properties (the health promotion effects in human and animal models) of many plant cultivars. Therefore, elucidating the relationship between the metabolome and the bioactivity of diverse cultivars could be a novel strategy for identifying the nutraceutical potential of various plant cultivars for functional food design.

All the traditional cardiovascular risk factors (dyslipidemia, arterial hypertension, hyperglycemia, and diabetes) are associated with endothelial dysfunction [14]–[16]. Thrombin is a protease produced on the surface of injured endothelium from prothrombin circulating in the blood. It alters endothelial permeability by stimulating cell contraction through reorganization of the cytoskeleton. This increases the size of intercellular gaps and allows entry of inflammatory cells and atherogenic lipoproteins [14]–[16]. A key event in the regulation of endothelial barrier function is actomyosin-driven contraction. Contraction of endothelial cells (ECs) is initiated by Thr-18/Ser-19 phosphorylation of the 20-kDa myosin regulatory light chain (MRLC), which is tightly associated with F-actin filament reorganization. Thrombin activity rapidly increases MRLC phosphorylation, stress fiber formation, and endothelial permeability. Thus, suppression of thrombin-induced MRLC phosphorylation in ECs may improve endothelial dysfunction and may prevent progression of cardiovascular diseases such as atherosclerosis.

Green tea has various health-promoting activities, and these activities vary from cultivar to cultivar. However, there is little information available for comparing numerous cultivars on the basis of their bioactivity. To properly utilize the nutraceutical properties of green tea, therefore, we need to clarify the relationship between cultivar and bioactivity. For nutraceutical evaluation, it is important to elucidate which cultivars have bioactivity, and which compounds contribute directly or indirectly to this bioactivity. In this study, we applied metabolic profiling techniques to evaluate the bioactivity of 43 representative cultivars of Japanese green tea (Table 1). The aim of our research was to evaluate the relationship between the metabolome and bioactivity (health promotion effect) of diverse tea cultivars. To test bioactivity we investigated the ability of leaf extracts to inhibit thrombin-induced MRLC in human umbilical vein endothelial cells (HUVECs), as a potential hallmark of vascular endothelial dysfunction. In addition, analyses of metabolic data from all tea extracts clearly discriminated green tea cultivars according to their bioactivity. Using regression analysis, we constructed a model to predict the bioactivity of tea cultivars on the basis of their metabolic data. These approaches comprise a useful strategy both for evaluation of bioactivity of green tea cultivars and for identification of bioactive factors.

**Table 1:** Forty-three kinds of the representative Japanese green tea cultivars.

No.	Cultivar	No.	Cultivar	No.	Cultivar
1	Seishin-oolong	16	Asagiri	31	Samidori
2	Fukumidori	17	Hokumei	32	Komakage
3	Benifuji	18	Asahi	33	Hatsumomiji
4	Minekaori	19	Sayamakaori	34	Ryoufuu
5	Benihikari	20	Meiryoku	35	Minamisayaka
6	Minamikaori	21	Kanayamidori	36	Saemidori
7	Benihomare	22	Yamatomidori	37	Okuyutaka
8	Izumi	23	Asatsuyu	38	Okumidori
9	Fuusyun	24	Toyoka	39	Yutakamidori
10	Tamamidori	25	Yaeho	40	Yabukita
11	Ohba-oolong	26	Ujihikari	41	Benifuuki
12	Seishintaipan	27	Ooiwase	42	Cha Chuukanbohon Nou-6
13	Kuritawase	28	Gokou	43	Sunrouge
14	Syunmei	29	Inzatsu131		
15	Sayamamidori	30	Surugawase		

## Materials and Methods

### Chemicals and antibodies

Epigallocatechin-3-*O*-gallate (EGCG) was obtained from DSM Nutritional Products (Amsterdam, Netherlands). Epicatechin-3-*O*-gallate (ECG), epigallocatechin (EGC), and epicatechin (EC) were obtained from Mitsui Norin Co. Ltd. (Tokyo, Japan). Thrombin, myricetin, quercetin-3-β-D-glucoside (Que-glu), anti-β-actin antibody, superoxide dismutase (SOD), and catalase were purchased from Sigma-Aldrich (St. Louis, MO, USA). Caffeine and quercetin (Que) were obtained from Nacalai Tesque Inc. (Kyoto, Japan). Theanin was purchased from Taiyo Kagaku (Yokkaichi, Japan). Theobromine was obtained from Wako Pure Chemical Industries, Ltd. (Osaka, Japan). Cyanidin (Cya), cyanidin-3-*O*-galactoside (Cya-gal), cyanidin-3-*O*-glucoside (Cya-glu), delphinidin (Del), delphinidin-3-*O*-glucoside (Del-glu), and quercetin-3-*O*-galactoside (Que-gal) were obtained from ExtraSynthese (Genay Cedex, France). Petunidin-3-*O*-glucoside (Pet-glu) was purchased from Tokiwa Phytochemical Co., Ltd (Sakura, Japan). Rabbit anti-phosphorylated MRLC (Thr-18/Ser-19) antibody was purchased from Cell Signaling Technology, Inc. (Danvers, MA, USA). Rabbit anti-MLC2 (FL-172) antibody (against whole MRLC) was obtained from Santa Cruz Biotechnology, Inc. (Santa Cruz, CA, USA). Horseradish peroxidase (HRP)-conjugated anti-rabbit IgG antibody was purchased from Rockland Immunochemicals Inc. (Gilbertsville, PA, USA). Cyanidin-3-*O*-(6-*O*-(E)-p-coumaroyl)-β-galactoside (Cya-cou-gal) was kindly provided from Asahi Breweries Ltd. (Moriya, Japan).

### Tea sample preparation

We analyzed 43 major Japanese green tea cultivars (Table 1), which are registered at and harvested from the National Institute of Vegetable and Tea Sciences, Japan. For each cultivar, dried leaf powder (200 mg) was added to 10 mL boiling water. The mixture was soaked for 10 min and stirred twice during this time. The extract was filtered through 90-µm filter paper (Advantec, Tokyo, Japan), and the filtrate was centrifuged at 1,680×*g* for 10 min. The supernatant was filtered through a 0.2-µm syringe filter (Sartorius Stedim Biotech, Goettingen, Germany). We used polyvinylpolypyrrolidone (PVPP: Sigma-Aldrich, St. Louis, MO, USA) as a polyphenol adsorbent to remove polyphenols from the solution. The PVPP (100 mg) was swollen in 1 mL H_2_O for 30 min. After centrifugation at 300×*g* for 5 min, the supernatant was removed and the precipitated gel pellets (300 µL) were mixed with a one-half volume of tea extract (150 µL). After centrifugation at 300×*g* for 5 min, the recovered supernatant was used as PVPP-treated tea extract.

### Cell culture and stimulation

Normal human umbilical vein endothelial cells (HUVECs) were obtained from Lonza (Basel, Switzerland) and were maintained in EGM-2 (Lonza) supplemented with 5% fetal bovine serum (FBS: Biological Industries, Kibbutz Beit Kaemek, Israel) in a humidified atmosphere with 5% CO_2_ at 37°C. The level of MRLC phosphorylation was determined by western blot analysis using specific antibodies against phosphorylated MRLC at Thr-18/Ser-19 and antibodies against whole MRLC. For this analysis, the cells were incubated at a density of 2×10^5^ cells/well in 24-well plates for 24 h. Then, the cells were treated with each 1% tea extract or each polyphenol (10 µM) in EGM-2 medium supplemented with 5 U/mL SOD and 200 U/mL catalase for 20 min. These antioxidant enzymes were added to decrease the pro-oxidant effect of green tea polyphenols. Then, thrombin (0.5 U/mL) was added and the cells were incubated for 5 min. This method comprises the modified and optimized conditions based on our previous report [17] to effectively evaluate the effect of tea extracts on thrombin-induced MRLC phosphorylation in HUVECs. The samples obtained were subjected to western blot analysis.

### Western blot analysis

**Figure 1:**
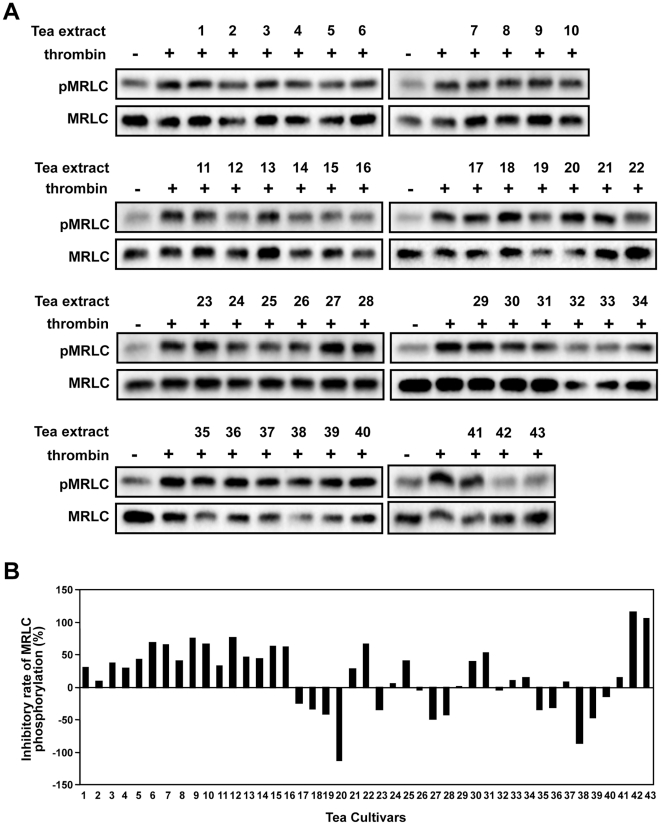
Effects of extracts from 43 Japanese green tea cultivars on thrombin-induced MRLC phosphorylation in HUVECs. **A**) After treatment of HUVECs with each 1% tea extract for 20 min, cells were stimulated with thrombin for 5 min, then lysed. Total cellular protein was subjected to western blot analysis using anti-phosphorylated MRLC (Thr18/Ser19) antibody. **B**) Inhibitory rate of MRLC phosphorylation (%) was calculated by comparing pMRLC/MRLC intensity in treated cells with that in non-treated cells (thrombin only).

After stimulation, the cells were lysed in cell lysis buffer containing 50 mM Tris-HCl (pH 7.5), 150 mM NaCl, 1% Triton X-100, 1 mM EDTA, 50 mM NaF, 30 mM Na_4_P_2_O_7_, 1 mM phenylmethanesulfonyl fluoride, 2 µg/mL aprotinin, and 1 mM pervanadate. Proteins were resolved on 10% SDS-polyacrylamide gels and then transferred onto a nitrocellulose membrane. The membranes were blocked with 2.5% bovine serum albumin and incubated with anti-phosphorylated MRLC (Thr-18/Ser-19) or anti-MLC2 antibody to evaluate phosphorylated or whole MRLC, respectively, followed by incubation with HRP-conjugated anti-rabbit IgG secondary antibodies. Epitopes on proteins specifically recognized by the antibody were visualized using the ECL Advance kit (GE Healthcare UK Ltd., Buckinghamshire, England). Band intensities were quantified using NIH Image-J software (Bethesda, MD, USA). Samples were analyzed in triplicate, and representative data are shown in figures. The relative band intensity (phosphorylated MRLC/whole MRLC: pMRLC/MRLC) of each sample was calculated using data from triplicate analyses. The results are shown as a percentage, calculated from the amount of pMRLC/MRLC in treated cells (thrombin + tea extract or single compounds) compared with that in non-treated control cells (+ thrombin only). Data shown are means ± SEM (*n* = 3). In Fig.1, the inhibitory effect of various tea extracts on MRLC phosphorylation is expressed as the rate of inhibition of MRLC phosphorylation (%). This was calculated by comparing the intensity of pMRLC/MRLC in each treatment with that in non-treated cells (+ thrombin only).

### LC-MS analysis

All extracts were subjected to high-performance liquid chromatography (HPLC) with time-of-flight MS (LC-MS) analysis using a LCMS-IT-TOF instrument (Shimadzu, Kyoto, Japan). For the PVPP test, both PVPP-treated and -untreated tea extracts (YB and SR) were analyzed. The instrument was fitted with a Luna 5u C18(2) 100A, 5 µm, 1.0×250 mm column (Phenomenex, Torrance, CA). The oven temperature was 40°C. The conditions of the mobile phase were as follows; linear gradient, consisting of solvent A, H_2_O (0.05% formic acid), and solvent B, methanol (0.05% formic acid). Solvent B was increased from 5% to 60% over 7.5 min, and further increased from 60% to 100% at 10.1 min, at flow rate of 0.1 mL/min. HPLC chromatograms of tea extracts (YB and SR) with or without PVPP treatment were obtained at UV 254 nm. The MS instrument was operated using an ESI source in positive ionization mode with survey scans acquired from *m/z* 70 to 700. Ionization parameters were as follows: capillary voltage, 4.5 kV; nebulizer gas flow, 1.5 L/min; CDL temperature, 200°C; heat block temperature, 200°C. Tea extracts were diluted 1∶10 with distilled water and 10 µM 4-hydroxybenzophenone (4-HB) was added as an internal standard. Samples were filtered through a 0.2-µm PTFE filter, and 3 µL was injected.

### Multivariate statistical analysis

**Figure 2:**
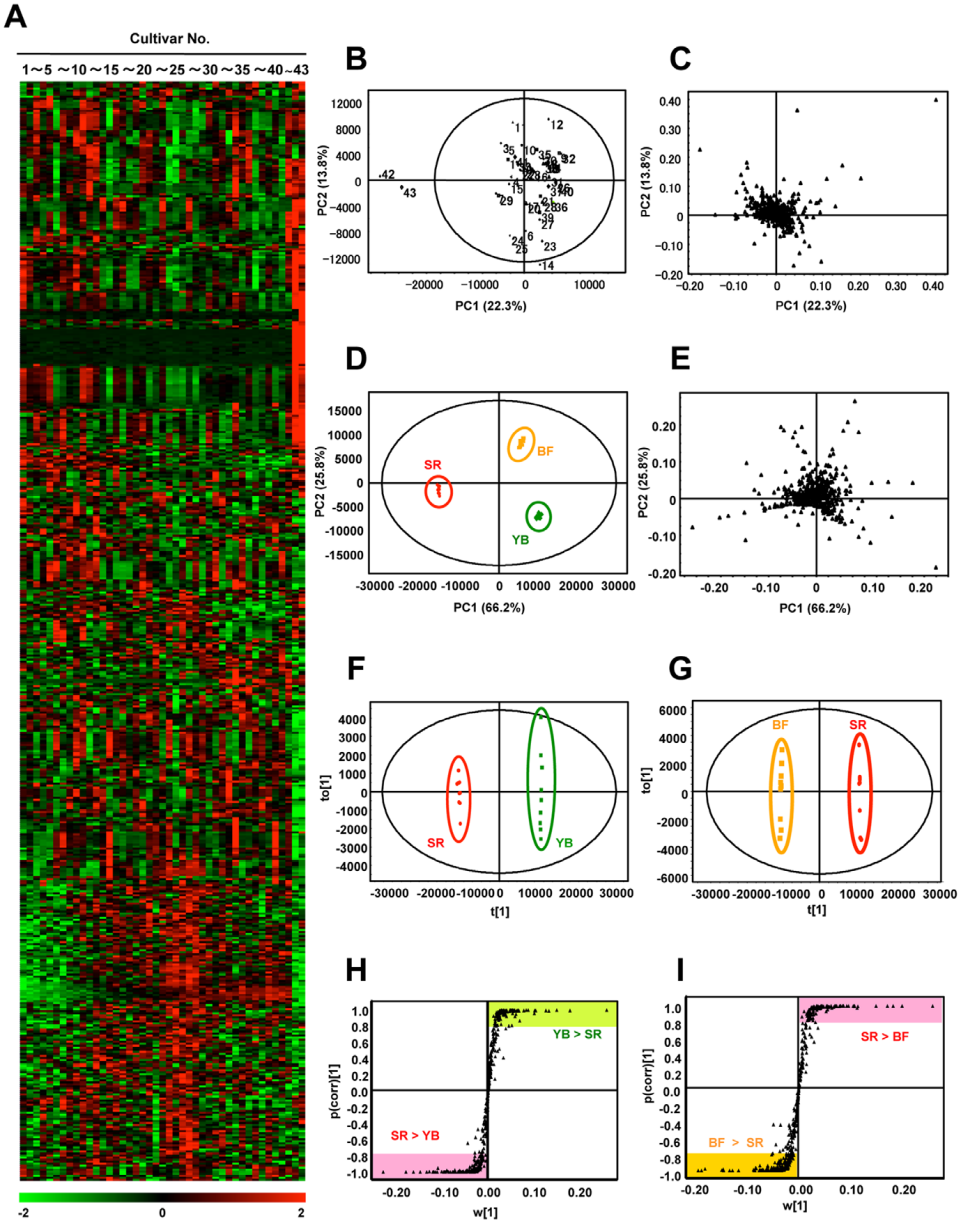
Multivariate statistical analysis of LC-MS metabolite profiles derived from various tea extracts. **A**) Heat map of diverse Japanese green tea extracts. Columns represent metabolic profile of single cultivars, rows represent 541 individual analytes. Colors correspond to relative metabolite areas. **B**) PCA score plot shows separate clustering of MS profiles corresponding to Nou-6 and SR, and other cultivars. **C**) Corresponding loading plots of all samples show MS peaks that differ among samples. **D**) PCA score plot derived from three representative tea cultivars; Yabukita (YB), Benifuuki (BF) and SR. **E**) Corresponding loading plots of three tea cultivars (YB, BF, and SR) show MS peaks that differ among samples. OPLS-DA score plots (**F** and **G**) and loading S-plots (**H** and **I**) were derived from each LC-MS data set (**F** and **H**: YB vs SR; **G** and **I**: BF vs SR). S-plot shows covariance w against correlation p (corr) of variables of the discriminating component of OPLS-DA model. Cut-off values for p (corr) < |0.8| were used to select metabolites that most strongly contributed to differences between two tea extracts.

**Figure 3:**
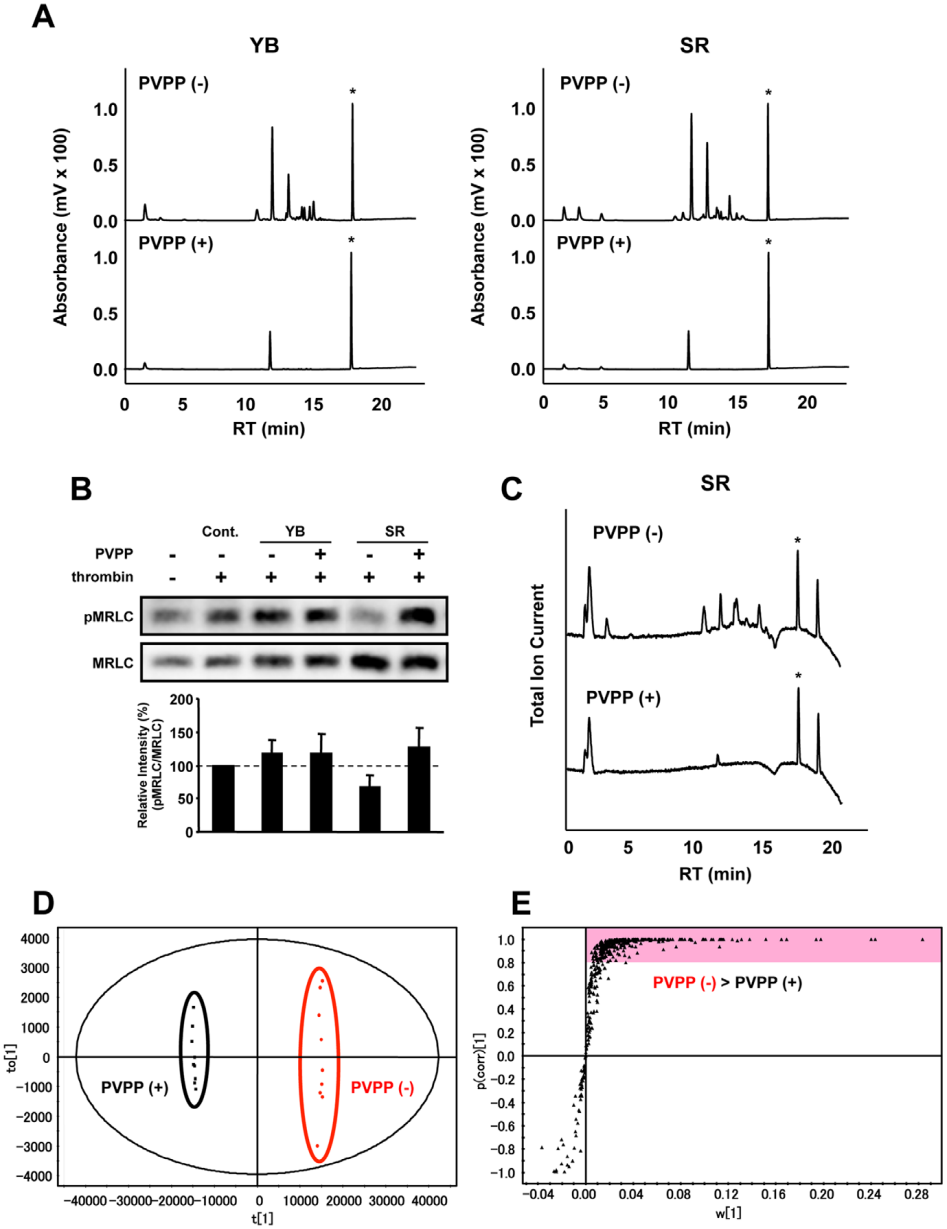
Effects of removing polyphenols on activity of tea extracts in MRLC phosphorylation and on metabolite profiles. **A**) HPLC-UV chromatograms of YB and SR extracts with or without PVPP treatment. Asterisk marks internal standard (4-HB). **B**) After treatment of HUVECs with each 1% tea extract for 20 min, cells were stimulated with thrombin for 5 min and then lysed. Total proteins were analyzed by western blot. Relative band intensity (pMRLC/MRLC) is expressed as a percentage, calculated from value in tea extract-treated cells compared with that in non-treated control cells (+thrombin). Values shown are means ± SEM (*n* = 3). **C**) Total ion current chromatograms of SR with or without PVPP treatment. Asterisk marks internal standard (4-HB). **D**) OPLS-DA score plots and **E**) loading S-plots derived from each LC-MS data set (SR vs SR+PVPP). S-plot shows the covariance w against the correlation p (corr) of variables of the discriminating component of OPLS-DA model. Cut-off values for the p (corr) < |0.8| were used to select metabolites that most strongly contributed to differences between two tea extracts.

For all LC-MS datasets, data were processed using the free software XCMS (http://masspec.scripps.edu/xcms/xcms.php) to extract and align peaks. Total tea extracts (43 tea cultivars; Fig. 1 and 2), tea extracts from three cultivars (YB, BF, and SR; Fig. 2), and two types of treated tea extracts (YB and SR with or without PVPP treatment; Fig. 3) were evaluated separately by multivariate statistical analysis. Generally, this analysis is used to clarify similarities and differences among samples on the basis of multivariate data (e.g., MS datasets). A multivariate approach can decrease the complexity of huge MS datasets, and can reveal relationships among samples or datasets. These relationships are usually displayed as scatter plots (score plots). Since hundreds of variables (peaks) are obtained in MS analyses, the relationships among samples must be theoretically interpreted on hundreds of dimensional axes (variables), but these relationships cannot be displayed simply. To visualize the features of samples, multivariate statistical analysis can extract features of samples by dimensional reduction. That is, hundreds of original variables are decreased to two or three synthetic variables, which are orthogonal with each other. This maximizes the statistical variance of samples, while leaving the original feature of samples largely unaffected [18]. The synthetic variables consist of hundreds of original variables. An understanding of the contribution of each original variable to the synthetic variables (loading plot) leads to the identification of key variables (compounds) that contribute to the relationships (similarity or difference) among samples. In this study, we carried out multivariate data analyses (PCA and OPLS-DA) [19] using SIMCA-P+ version 12.0 (Umetrics, Umea, Sweden). PCA models are depicted as score plots and consist of two synthetic variables: principal component (PC) 1 (the greatest variance of data) and PC2 (the second greatest variance of data, orthogonal with PC1). These display intrinsic groups of samples based on spectral variations. The corresponding loading plots show the contribution of each spectral variable to score formation. Therefore, this analysis can explain the original feature of samples based on the ratio of the sum of percentages of PC1 and PC2. All variables obtained from LC-MS datasets were mean-centered and scaled to Pareto variance [19]. The quality of OPLS-DA models was evaluated by the goodness-of-fit parameter R^2^ and the predictive ability parameter Q^2^. R^2^ and Q^2^ values higher than 0.5 indicated good quality of OPLS-DA models. Metabolite peaks were assigned by MS/MS analysis or by searching their accurate masses using online metabolite databases (KEGG; http://www.genome.jp/kegg/, METLIN; http://metlin.scripps.edu/, MassBank; http://www.massbank.jp/).

PLS, PLS-orthogonal signal correction (OSC), and OPLS were chosen to create the prediction model. PLS, which can be described as the regression extension of PCA, was calculated using SIMCA-P+. PLS derives latent variables that maximize the covariation between measured metabolite data and the response variable (inhibitory activity) regressed against. This differs from PCA, which utilizes the maximum variation in the metabolite data matrix. OSC is normally used to remove uncorrelated variables or those orthogonal to inhibitory activity from metabolite data using the nonlinear iterative partial least-squares algorithm.

A heat map was generated the statistical package MultiExperiment Viewer (MeV v4.6.1) (http://www.tm4.org/mev/). This summarizes the Z-score values of 541 peaks, which shows differences in metabolite profiles among cultivars.

## Results

### Effects of Japanese green tea extracts on thrombin-induced phosphorylation of MRLC in HUVECs

Here, we examined the effects of extracts from 43 cultivars of Japanese green tea (Table 1) on thrombin-induced MRLC phosphorylation in HUVECs by western blot analysis. We used aqueous extracts to determine bioactivity and composition, because this is the form in which green tea is consumed. As shown in Table 2, the 43 cultivars showed diverse effects. Some inhibited MRLC phosphorylation (Fig. 1A, B), especially Cha Chuukanbohon Nou-6 (Nou-6) and Sunrouge (SR). Some enhanced MRLC phosphorylation, especially Meiryoku and Okumidori. Several cultivars did not affect the level of MRLC phosphorylation, e.g., Inzatsu131, Ujihiraki, and Komakage. Thus, there were differences among cultivars in their ability to regulate thrombin-induced MRLC phosphorylation. The different compositions of these extracts were expected to underlie cultivar-specific bioactivity.

**Table 2:** Ranking of inhibitory rates of 43 Japanese green tea cultivars on thrombin-induced phosphorylation of MRLC in HUVECs.

Rank	Cultivar	Inhibitory rate (%)	No.	Rank	Cultivar	Inhibitory rate (%)	No.
1	Nou-6	115.9	42	23	Ryoufuu	14.4	34
2	Sunrouge	104.8	43	24	Benifuuki	14.3	41
3	Seishintaipan	75.8	12	25	Hatsumomiji	9.6	33
4	Fuusyun	74.4	9	26	Fukimidori	8.4	2
5	Minamikaori	67.6	6	27	Okuyutaka	7.1	37
6	Tamamidori	66.0	10	28	Toyoka	5.5	24
7	Yamatomidori	65.5	22	29	Inzatsu131	1.0	29
8	Benihomare	64.2	7	30	Komakage	−4.1	32
9	Sayamamidori	62.3	15	31	Ujihikari	−4.4	26
10	Asagiri	60.8	16	32	Yabukita	−13.9	40
11	Samidori	52.3	31	33	Hokumei	−24.5	17
12	Kuritawase	45.4	13	34	Saemidori	−31.3	36
13	Syunmei	43.2	14	35	Asahi	−32.6	18
14	Benihikari	42.0	5	36	Minamisayaka	−33.6	35
15	Yaeho	40.3	25	37	Asatsuyu	−34.8	23
16	Izumi	39.4	8	38	Sayamakaori	−41.0	19
17	Surugawase	38.2	30	39	Gokou	−42.3	28
18	Benufuji	37.1	3	40	Yutakamidori	−46.8	39
19	Ohba-oolong	32.6	11	41	Ooiwase	−49.2	27
20	Seishin-oolong	29.5	1	42	Okumidori	−85.4	38
21	Minekaori	28.3	4	43	Meiryoku	−112.6	20
22	Kanayamidori	27.9	21				

### LC-MS-based metabolic profiling to evaluate bioactivity of Japanese green tea

Aqueous crude extracts of tea leaves from the 43 cultivars were subjected to LC-MS to investigate differences in their compositions. In analyses of complex mixtures such as crude extracts, two or more compounds can be co-eluted. The obtained complex spectral data are usually processed to extract and align peaks. We extracted 541 peaks from a complex chromatogram and used multivariate statistical analysis to decrease the complexity of the spectra datasets. This chemometric approach has the potential for use in classification and bioactivity assessment without any prepurification methods such as extraction of arbitrary constituents from crude extracts prior to LC-MS measurement. To provide comparative interpretations and to visualize metabolic differences among cultivars in relation to their bioactivity, we analyzed the LC-MS spectra datasets using several multivariate analyses (see detailed information about multivariate statistical analyses in Materials and methods).

Heat map analysis provides an overview of all observations or samples in a dataset by highlighting holistic differences in the complex metabolic data. This method can be used to visualize simultaneously the metabolic profiles of many cultivars. As shown in Fig. 2A, the metabolic profiles clearly differed among green tea cultivars. The differences in chemical composition among cultivars may be responsible for differences in their bioactivity. Thus, we conducted further experiments to determine which analytes were responsible for variations in bioactivity.

Another unsupervised multivariate analysis method, the PCA model, provides an overview of all observations or samples in a dataset [18]. Groupings, trends, and outliers can also be found. Unlike the heat map analysis, this model can visualize the relationships among samples on a two dimensional model plane. The PCA score plot showed clear independent clusters, one consisting of cultivars with higher bioactivity (Nou-6 and SR), and the other consisting of the remaining cultivars (Fig. 2B). In the corresponding loading plot (Fig. 2D), several metabolites, such as EC, EGC, ECG, EGCG, caffeine, theanin, myricetin, theogallin, and other non-assigned *m/z* peaks had a comparatively strong impact on the clear separation of each cluster along the principal component axes (PC 1 and PC 2). In particular, theanin and caffeine strongly contributed to the separation of groups along PC1, and theogallin contributed to the separation of groups along PC2. To further explore the metabolic differences among tea cultivars, we performed another PCA analysis using three representative tea cultivars: the non-bioactive cultivar Yabukita (YB), the bioactive cultivar SR, and the less bioactive cultivar Benifuuki (BF). YB is the most commonly consumed and widely distributed cultivar in Japan, accounting for 70−80% of all green tea consumed. In the bioactivity assay, YB was ranked 32/43 (inhibitory rate <0), SR was ranked 2/43 (inhibitory rate >100), and BF was ranked 18/43 (0<inhibitory rate<50). BF was also selected because it has reported biomedical activities in human models [20], [21]. The PCA score plot showed a clear independent cluster formation (Fig. 2C), and the distribution of the three tea cultivars was relatively similar to that observed among the 43 cultivars (Fig. 2E).

Although the PCA model provided an overview of all observations or samples, the details of differences in each cluster remained unclear. The supervised method, OPLS-DA, was then used to isolate the variables responsible for differences among the three representative tea cultivars. The OPLS-DA score plots are shown in Fig. 2F and 2H. The goodness-of-fit parameter R^2^ and the predictive ability parameter Q^2^ were 0.926 and 0.999, respectively (YB vs SR), or 0.921 and 0.999, respectively (BF vs SR). These results indicated that the OPLS-DA models were reliable. The OPLS-DA loading S-plot, a plot of the covariance versus the correlation in conjunction with the variable trend plots, allows easier visualization of the data. The variables that changed most significantly are plotted at the top or bottom of the ‘S’ shape plot, and those that do not vary significantly are plotted in the middle [19]. OPLS-DA S-plots for YB vs SR and BF vs SR are shown in Fig. 2G and 2I, respectively. Values for *p* (corr) > |0.8| were used to select variables that strongly contributed to the difference between the two cultivars. YB > SR was 116 peaks, YB < SR was 200 peaks, BF > SR was 158 peaks, and BF < SR was 145 peaks. As shown on the S-plot, Cya-glu, Cya-gal, and theobromine contributed to the differences between cultivars. These peaks could not be found in the PCA score plot, indicating that OPLS-DA can provide valuable information for clarifying which factors are responsible for differences among cultivars.

### Involvement of polyphenols in bioactivity of Japanese green tea extract

Among green tea constituents, polyphenols are the most abundant and most active components for inhibiting diseases and related reactions. To examine whether polyphenols are involved in the inhibition of thrombin-induced MRLC phosphorylation by tea extracts, we removed polyphenols from samples using the polyphenol adsorbent PVPP. HPLC-UV chromatograms from the non-bioactive tea cultivar YB and highly bioactive tea cultivar SR are shown in Fig. 3A. The decreased sizes and disappearance of many peaks showed that various polyphenols were effectively removed from these extracts. We determined the effects of these PVPP-treated tea cultivars on thrombin-induced MRLC phosphorylation in HUVECs (Fig. 3B). PVPP treatment did not affect the activity of YB. However, it prevented the activity of SR, suggesting that polyphenols have an important role in the bioactivity of SR. To further explore the bioactive components of SR, samples of SR and PVPP-treated SR were analyzed by LC-MS. PVPP treatment resulted in a decrease or disappearance of many peaks from the total ion current chromatogram (Fig. 3C). The affected peaks were identified by analyzing LC-MS metabolite peak profiles from SR and PVPP-treated SR tea extracts by OPLS-DA. In the score plot, the independent cluster formation between both samples, with R^2^ (0.999) and Q^2^ (0.999), showed that the OPLS-DA model was statistically significant (Fig. 3D). This observation indicates that this statistical model can discriminate the differences among metabolite profiles based on PVPP treatment. The loading S-plot can visually extract variables (metabolite peaks) contributing to the differences between SR and PVPP-treated SR (Fig. 3E). Values for *p* (corr) >0.8 were used to select variables that strongly contributed to differences between the two extracts. In total, 359 peaks were decreased or removed by PVPP treatment, suggesting that some of these peaks are bioactive components.

**Figure 4:**
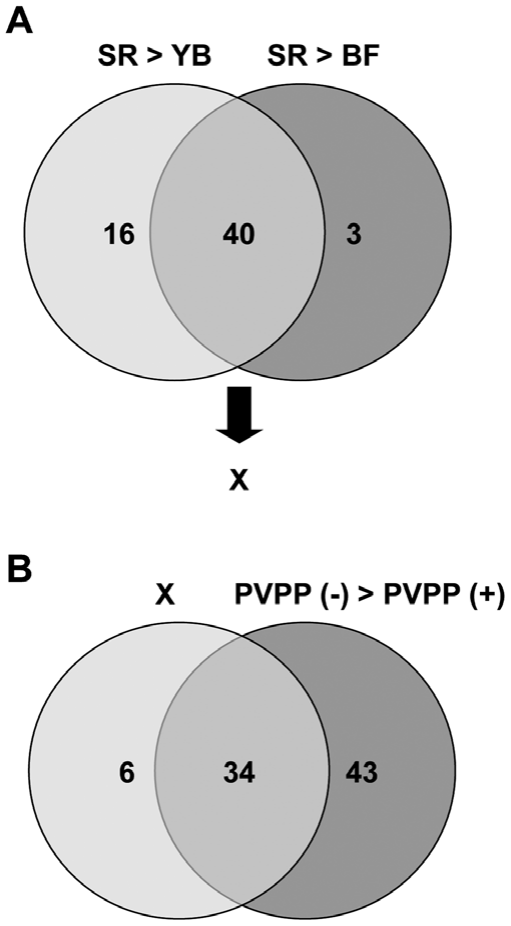
Decreasing number of candidate metabolites contributing to bioactivity of tea extracts. We used variables corresponding to p (corr) > |0.8| and the w > |0.05| in OPLS-DA loading S-plot to identify peaks most strongly contributing to differences among cultivars. Venn diagram illustrating the proportion of unique and overlapping ion features in **A**) extracts from YB, BF and SR, and **B**) SR extracts with or without PVPP treatment.

**Figure 5:**
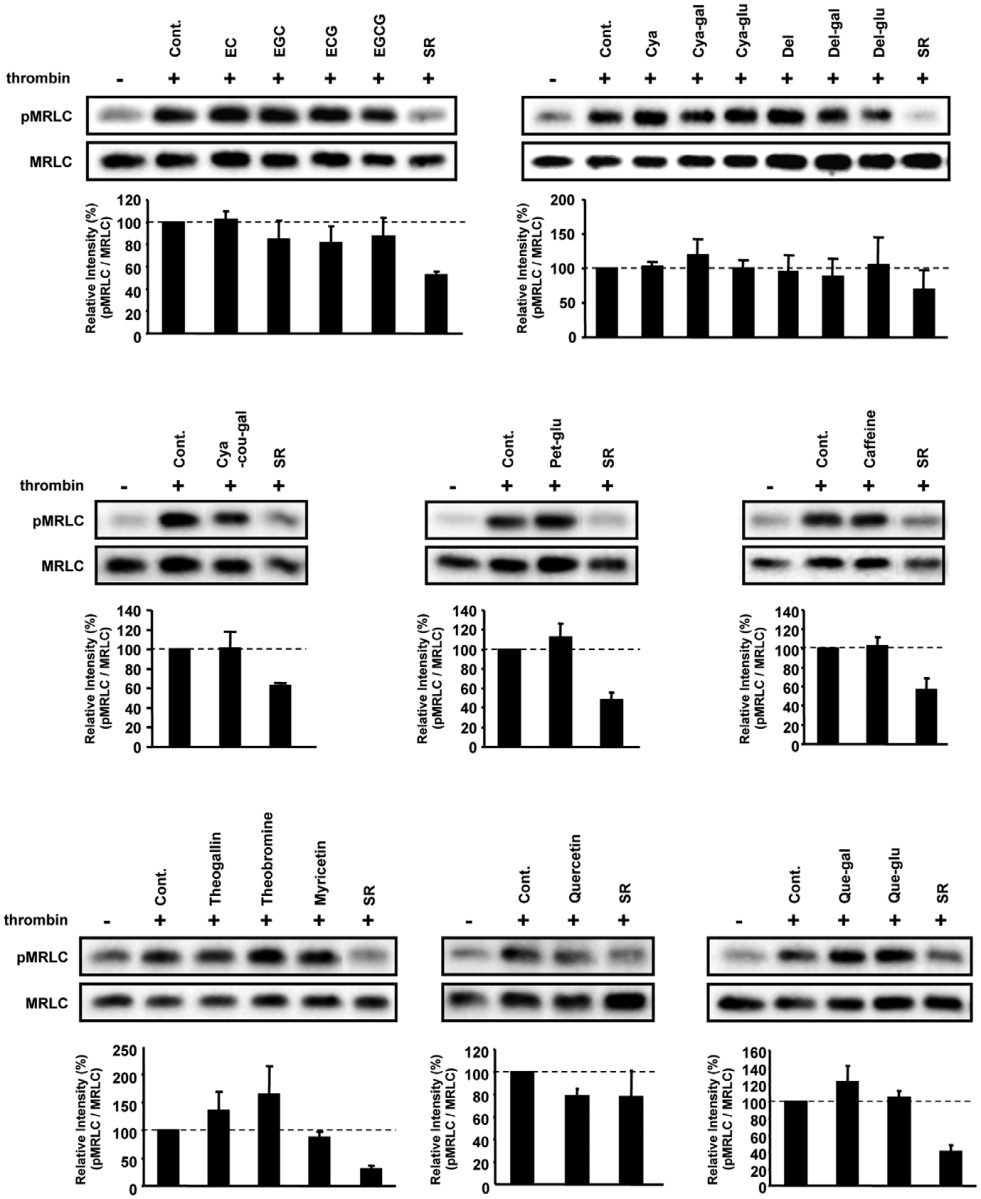
Effect of the representative tea constituents and several identified metabolites on MRLC phosphorylation. After treatment of HUVECs with each tea constituent at the concentration of 10 µM for 20 min, the cells were stimulated with thrombin for 5 min, then lysed. Total cellular proteins were analyzed by western blot. Relative band intensity (pMRLC/MRLC) is expressed as a percentage, calculated from value in treated cells compared with that in non-treated control cells (+thrombin). Values shown are means ± SEM (*n* = 3).

Taken together with this finding (Fig. 3) and the above-mentioned PCA and OPLS-DA results (Fig. 2), many metabolite peaks were possible candidates for contributing to bioactivity of SR extracts. To decrease the number of metabolite peaks, we selected some metabolite peaks from OPLS-DA under the following constraints: in S-plots, we used cut-off values for *p* (corr) <|0.8| and *w*<|0.05| to select metabolites that strongly contributed to the differences between the two cultivars. A Venn diagram illustrating the proportion of unique and overlapping ion features in the three different tea extracts is shown in Fig. 4. Among a total of 502 metabolite peaks, 56 were more abundant in SR than YB, and 43 were more abundant in SR than in BF (Fig. 4A). Forty peaks overlapped between SR > YB and SR > BF groups. Intriguingly, almost all of these peaks, 34/40 (85%), overlapped with the data of SR > SR+PVPP (Fig. 4B). Among these 34 peaks (Table S1) and the 29 peaks derived from PCA (Table S2), the representative peaks contributed to the formation of the independent cluster consisting of YB, BF, and SR in the PCA score plot. The representative peaks were assigned by MS/MS analysis or by searching their accurate masses using online metabolite databases. Chemical structures of nine types of identified metabolites, including anthocyanins, flavonols, and galloyl glucose are shown in Fig. S1. Generally, many bioactivities of glycosides depend on the structure of the aglycon. We examined the effects of identified glycosides and their aglycons as well as major green tea constituents, catechins, flavonols, and methylated xanthines, which were also observed in the PCA loading analysis, on the thrombin-induced MRLC phosphorylation in HUVECs (Fig. 5). None of these compounds inhibited MRLC phosphorylation. This result suggests that other unidentified compounds and compounds excluded by the narrowing procedure (Fig. 4), and/or combinations of compounds may be involved in bioactivity.

### Bioactivity-prediction model

**Figure 6:**
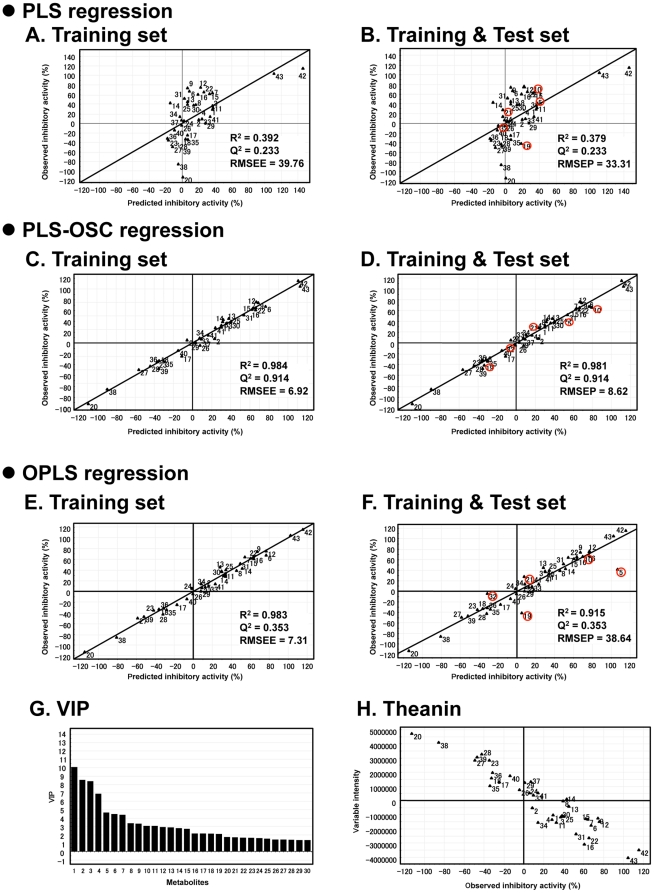
Observed and predicted activity of 43 green tea cultivars, using three regression models. **A**, **B**) PLS model, **C**, **D**) PLS model with OSC preprocessing method, and **E**, **F**) OPLS model. Models were calculated from LC-MS data set of (A, C, E) 38 tea samples as the training set and B, D, F) 43 tea samples included in training and test (red circle) sets. **G**) Bar chart showing influence of variables used to create bioactivity predictor for green tea cultivars (Y-axis is value of variable importance in the projection, VIP). **H**) Theanin, the metabolite with the highest VIP value, was significant for creating the bioactivity prediction model for tea cultivars.

To determine whether bioactivity of the tea cultivars was correlated with their metabolic profiles, we created a bioactivity prediction model based on regression analysis. To obtain the regression, a mathematical model is created based on the system behavior, and then optical values for model parameters are determined with respect to training samples [22]. Then, values of unknown independent values are predicted using the resulting training model [22]. We used PLS or OPLS regressions, which are chemometric projection methods relating two independent variables via a linear multivariate model, to predict the bioactivity of tea cultivars. The predicted inhibitory activity was calculated from the peak intensity of each metabolite (Table S3). The entire dataset from 43 samples was divided into two parts: 38 training set samples used to create the model, and five test set samples (sample no. 5, 10, 19, 21, and 32). When all 43 samples were ranked according to bioactivity, these samples corresponded to every eighth sample, and were used to verify the model's predictive ability. They were not included in the regression model. The PLS or OPLS relationship between measured and predicted inhibitory activities of green tea cultivars is shown in Fig. 6. The quality of the regression model can be verified by the correlation coefficient R^2^ and the cross-validated correlation coefficient Q^2^, as well as the validation errors of estimation and that between measured and predicted values; these are known as root mean squared error of estimation (RMSEE) and root mean squared error of prediction (RMSEP), respectively. Generally, R^2^, which describes how well the data of the training set is mathematically reproduced, varies between 0 and 1, where 1 indicates a perfect fit between the model and the data. A prediction model is considered to be good when Q^2^ >0.5, and excellent if Q^2^ >0.9. The PLS regression model showed R^2^ = 0.392 and Q^2^ = 0.233 with RMSEE = 39.76 (Fig. 6A), indicating a poor fit and poor prediction ability. The ability of the model to predict the bioactivity of green tea cultivars was tested by entering the test set values into the PLS regression (Fig. 6B). The result was rather scattered from the ideal diagonal with RMSEP  = 33.31. The large validation error (33.31) was likely due to uncorrelated metabolite data variables interrupting the prediction of bioactivity variables, hence distorting the predictability of the model.

The quality of PLS regression can be improved by simplifying the complexity of variations using an orthogonal signal correction (OSC) approach. This decreases the number of variables in the metabolite data matrix by removing those that are linearly unrelated (orthogonal) to the bioactivity matrix [18]. By OSC processing of the PLS model, the linearity (R^2^) was improved by 251% (0.984/0.392), and the predictability was also improved (Q^2^ increased from 0.233 to 0.914; Fig. 6C). The cross-validation of the PLS-OSC regression model was performed using a test set as described above (Fig. 6D). The RMSEP value significantly decreased from 33.31 to 8.62. Both the increase of Q^2^ and the decrease of RMSEP indicated that the power of the predictive model was drastically improved by removing unwanted variations by signal correction. This meant that OSC was an effective filtering method to remove the anticipated variables and enhance the accuracy of the regression model.

OPLS is a modified version of ordinary PLS. This model may be built with one PLS-factor, and thus is much simpler than the corresponding PLS model [18]. OPLS was applied to the regression model for predicting bioactivity of green tea. The regression is shown in Fig. 6E and 6F, with R^2^ and Q^2^ values of 0.983 and 0.353, respectively. However, the predictive ability of the OPLS regression model did not improve, as the value of RMSEP increased from 33.31 to 38.64.

By comparing the R^2^, Q^2^, and RMSEP values of the three regression models, we determined that the best bioactive predictive model (that is, the one with the highest prediction accuracy) was that obtained from the PLS-OSC regression. In this model, variables that were highly relevant for explaining predicted bioactivity were also identified from VIP (variable importance in the projection) values (Fig. 6G). Large VIP values (>1) are the most relevant for explaining predicted bioactivity. Forty-six metabolite peaks showed VIP values greater than 1 (Table S4). Theanin (chemical structure; Fig. S2) showed the highest VIP value. Among the 46 peaks, eight (Cya-glu, Cya-gal, Cya-cou-gal, Del-glu, Del-gal, Que-glu, Que-gal, and theogallin) were identical to the selected peaks obtained from narrowing analysis of OPLS-DA data (Fig. 4). These eight peaks were relatively abundant in the cultivar SR, which showed higher bioactivity. The relative level of theanin was strongly inversely correlated with bioactivity (Fig. 6H). The cultivar SR, which had higher bioactivity, had lower levels of theanin than the non-bioactive cultivar YB. These observations suggest that theanin may act as a possible negative regulator of bioactivity in tea cultivars.

**Figure 7:**
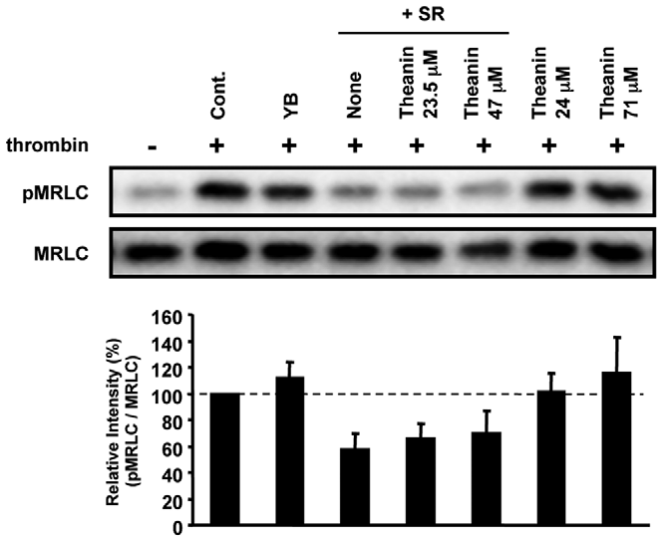
Combined effects of SR extract and theanin on thrombin-induced MRLC phosphorylation. HUVECs were pretreated with 1% YB or SR extract with or without theanin at indicated concentration for 20 min. Then, the cells were treated with thrombin for 5 min and lysed. Total cellular proteins were analyzed by western blot. Phosphorylation levels of MRLC were normalized to MRLC. Relative band intensity (pMRLC/MRLC) is expressed as a percentage, calculated from value in treated cells compared with that in non-treated control cells (+thrombin).

To further elucidate the potential role of theanin in the bioactivity of tea, we examined its effects on the ability of SR to inhibit MRLC phosphorylation in HUVECs (Fig. 7). We added 47 µM theanin to SR extract (containing 24 µM theanin) to achieve the same concentration as that in YB extract (71 µM). This addition of theanin decreased the inhibitory rate of SR extract from 42% to 30%. Although addition of theanin only weakly inhibited the bioactivity of SR, this result suggested that theanin could negatively regulate SR action and this could partially contribute to the non-bioactive properties of YB.

### Combined effects of identified metabolites in cultivar YB

**Figure 8:**
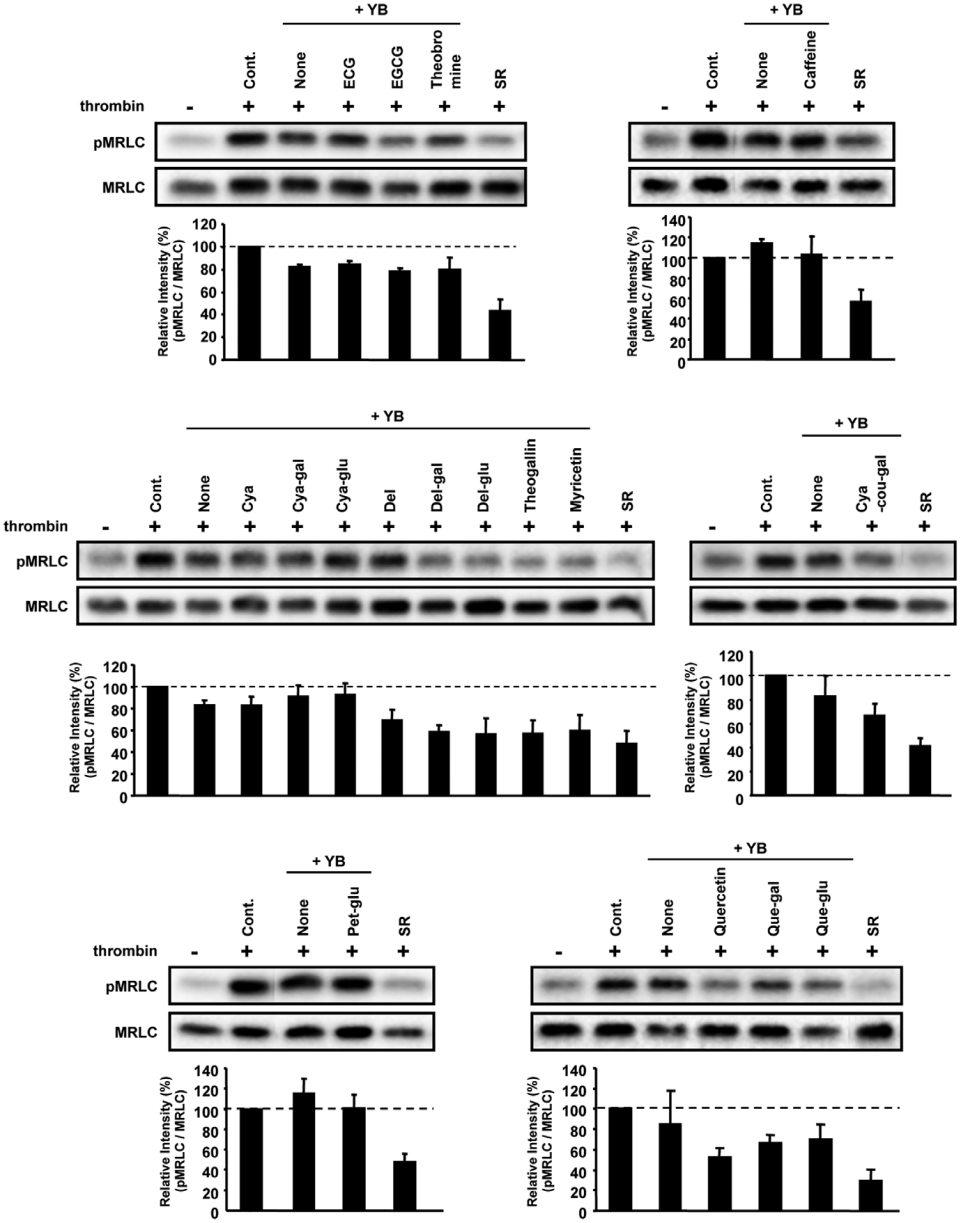
Combined effects of YB extract and identified metabolites on thrombin-induced MRLC phosphorylation. HUVECs were pretreated with 1% SR or YB extract with or without each indicated compound at 10 µM for 20 min. Then, cells were treated with thrombin for 5 min, then total proteins were extracted and analyzed by western blot. Phosphorylation levels of MRLC were normalized to MRLC. Relative band intensity (pMRLC/MRLC) is expressed as a percentage, calculated from value in treated cells compared with that in non-treated control cells (+thrombin).

While individual compounds may account for some bioactivity, a combination of compounds might be more effective with respect to the bioactivity of tea. Although successive OPLS-DA (Fig. 4) and regression analysis (Fig. 6) revealed several tens of important metabolite peaks, many of these peaks remained unassigned. Elucidating the bioactivity of each identified metabolite in the presence of numerous known and unknown constituents in tea extracts is an important approach for understanding the bioactivity of green tea and its effective nutraceutical applications. In that sense, an investigation on combinations of certain identified metabolites in non-bioactive tea extract could help to clarify the effect of each metabolite in a mixture of known and unknown tea constituents. In this study, we conducted combination tests in which we added 17 different compounds to extracts of the non-bioactive cultivar, YB. The 17 different compounds are shown in Fig. 5 (ECG, EGCG, Cya, Cya-gal, Cya-glu, Del, Del-gal, Del-glu, Cya-cou-gal, Pet-glu, caffeine, theogallin, theobromine, myricetin, Que, Que-gal, and Que-glu). Nine of these were metabolites with higher VIP values (theogallin, caffeine, Cya-gal, Cya-glu, EGCG, ECG, theobromine, Del-gal, Del-glu). When added to the non-bioactive tea extract, Del, Del-glu, Del-gal, Que, Que-glu, Que-gal, myricetin, theogallin, and Cya-cou-gal (chemical structures shown in Fig. S3) inhibited thrombin-induced MRLC phosphorylation (Fig. 8). Thus, these compounds were able to transform the non-bioactive YB extract into a bioactive extract.

## Discussion

Metabolomic analyses of plants have been used to study genotype, production origin, manufacturing type, sensory evaluation, cultivation method, climatic variables, and postfermentation year [6], [9]–[12], [23]–[28]. However, little is known about the relationship between bioactive function (health promotion effect in human and animal models) and numerous cultivars in a single plant species. Here, we have demonstrated for the first time that a metabolomics approach can be used to evaluate the bioactivity of various Japanese green tea cultivars and to identify bioactive factors. These new findings highlight the potential applications of metabolic profiling techniques to evaluate nutraceutical properties of diverse plant cultivars and foods, and thus propose a novel strategy for functional food design or drug discovery. Interestingly, a new Japanese green tea cultivar, Sunrouge (SR), showed some potential to improve endothelial dysfunction by suppressing thrombin-induced MRLC phosphorylation. Furthermore, this metabolomic approach allowed us to identify several bioactive factors that are enriched in SR. If introduced into non-bioactive lines, such factors may be capable of transforming non-bioactive cultivars into bioactive ones.

Recently, Ku and coworkers used metabolomic approaches (PCA, OPLS-DA, or PLS regression analysis) to determine the effect of manufacturing type or cultivation method on chemical composition of a single tea cultivar (green tea or pu-erh tea) [26], [27]. They suggested that several polyphenolic compounds were associated with manufacturing type, cultivation method, or antioxidant activity. In contrast, here, we investigated relationships between metabolomic data and health promotion effects (inhibitory effect on MRLC phosphorylation in HUVECs) in 43 green tea cultivars. In the cultivar SR, certain polyphenolic constituents (Del-glu, Del-gal, Que-glu, Que-gal, theogallin, and Cya-cou-gal) were associated with bioactivity. Although polyphenols have many health promotion effects, the relationship between these compounds and the inhibition of MRLC phosphorylation in human endothelial cells remains unclear. These polyphenols differed from those reported by Ku et al [26], [27]. In addition, such polyphenols, especially anthocyanins, were barely present in the most consumed and distributed Japanese green tea cultivar. These facts support the potential value of our identified polyphenols, and indicate that a metabolomic approach is a useful tool for identifying unique bioactive factors. In this study, we created a bioactivity-predictive PLS regression model using 43 Japanese green tea cultivars (Fig. 6). These analyses yielded unique lists of bioactivity-correlated constituents (46 peaks) in tea extracts. In future, such information may be useful for the development of markers to produce new cultivars with greater bioactivity, and to screen for bioactive tea cultivars. Furthermore, we can predict the potential bioactivity of numerous tea cultivars by analyzing their metabolomic data with our regression model, without the requirement for additional bioactivity assays.

In this study on bioactivity of 43 green tea cultivars, Nou-6 showed the highest activity for inhibiting thrombin-induced MRLC phosphorylation in HUVECs (Fig. 1). Previously, we reported that Nou-6 inhibited thrombin-induced MRLC phosphorylation in the rat aortic smooth muscle cell line A7r5. We suggested that this effect may provide an approach to suppress stress-induced contraction of blood vessels [17]. In addition, Nou-6 was found to contain high levels of anthocyanins. Recently, it was reported that intake of anthocyanins and anthocyanin-rich foods had a therapeutic effect against cardiovascular diseases [29]. In our most recent study, a crossover trial in healthy human volunteers showed that drinking of Nou-6 tea infusion reduced video display terminal work-induced visual fatigue and stress, as compared with YB (unpublished data). This unique cultivar is also rich in theogallin, one of the bioactive factors that inhibited thrombin-induced MRLC phosphorylation in HUVECs (Fig. 8) and a novel inhibitor of IgE production as a promising anti-allergic target [30]. These observations suggest that Nou-6 is an attractive green tea cultivar for ameliorating effects of stress and vascular function or for anti-allergic effects. However, this is a low-yielding cultivar, and it is difficult to grow. To overcome these problems, a new tea cultivar, Sunrouge (SR), was generated by natural crossbreeding of Nou-6. This novel cultivar contains high levels of anthocyanins, grows vigorously, is high yielding and easy to grow, and shows high resistance to anthracnose and gray blight. Interestingly the bioactivities and metabolotypes of Nou-6 and SR are very similar (Fig. 1, 2A, 2B). In the PCA score plot (Fig. 2B), Nou-6 and SR (no. 42 and 43, respectively) formed a group that was separate from the other 41 cultivars. In this plot (Fig. 2B, 2D), the confidence interval is defined by the confidence ellipse (95% confidence interval), and observations outside the confidence ellipse are considered outliers. If a sample shows a remarkably different metabolic profile from that of other samples it will fall outside the confidence ellipse. In the case of Nou-6 and SR, we repeatedly obtained similar results. This reproducibility suggested that Nou-6 and SR were unique cultivars with interesting compositional patterns, rather than outliers. We selected the SR cultivar for focused PCA analyses to elucidate detailed metabolic difference among tea cultivars (Fig. 2D, 2F–I). This was because of its interesting composition, but also because of its cultivation properties. The OPLS-DA results (Fig. 2) suggested that there were many differences in metabolites between SR and YB/BF. Almost all of the metabolite peaks focused by the two OPLS-DA (SR vs YB & SR vs YB) overlapped with those removed by PVPP treatment (Fig. 4). Considering the significance of the PVPP test to indicate bioactive groups (polyphenols) in SR tea extract (Fig. 3), it may be reasonable to use OPLS-DA for simple and high-precision screening for bioactive factors, without the need for additional experiments such as PVPP tests. These observations support the applicability of metabolic profiling with multivariate statistical analysis in nutraceutical research. Although some problems need to be solved, further metabolomic analyses and bioassays will increase our knowledge of the health promotion effects of the novel cultivar SR. In previous metabolomic research on tea, almost all analyses focused on differences among production regions, and little was known about biochemical differences among cultivars. Considering this fact and our findings (Fig. 1), functional studies on various green tea cultivars may expand the nutraceutical potential of green tea.

At present, human studies suggest that consumption of green tea can reduce the risk of cardiovascular diseases such as atherosclerosis. However, the mechanisms underlying this, including direct involvement in MRLC phosphorylation, and the differences in bioactivity among various green tea cultivars remain unclear [31]–[33]. Endothelial dysfunction is an early step in the development of atherosclerosis, and is associated with cardiovascular risk factors [14]–[16]. Enhancement of MRLC phosphorylation increases contraction and permeability of ECs, and therefore promotes dysfunction of the endothelial barrier during atherogenesis. Here, we showed for the first time the ability of numerous green tea cultivars to inhibit MRLC phosphorylation in HUVECs (Fig. 1). Although further animal and human studies are required, these results suggest that intake of green tea may help to prevent cardiovascular diseases such as atherosclerosis via a novel therapeutic target, i.e., the inhibition of MRLC phosphorylation. A combination of certain SR-specific polyphenols with an extract from the non-bioactive cultivar YB inhibited MRLC phosphorylation (Fig. 8). The fact that certain constituents identified by metabolic profiling can change non-bioactive extracts into active ones suggests that this approach could be used to expand the function and utilization of some tea cultivars. To further elucidate this bioactivity-inducing mechanism, we are now investigating various combinations of single tea constituents to clarify the molecular mechanism underlying the bioactivity of SR.

In the field of nutraceutical research, there are many reports on screening of plants and foods for positive bioactivity. However, there is little information about negative bioactive regulators. In addition to positive regulators in bioactive plants and foods, elucidation of negative regulators and the unique concomitant factors would be useful for the design of nutraceutical products for use in effective and safe functional foods. In that sense, combining metabolic profiling methods (PCA, OPLS-DA, and PLS regression analysis) with bioassays can yield information on both positive and negative bioactive compounds as mentioned above. In fact, several SR-specific biofactors were also found to be able to activate the non-bioactive cultivar YB (Fig. 8). This discovery provides a promising strategy to explore bioactivity-enhancing combinations of green tea and foods with abundant YB-activating biofactors (Del, Del-glu, Del-gal, Que, Que-glu, Que-gal, myricetin, theogallin, or Cya-cou-gal). Although theanin has been reported to have many health promotion effects, we observed for the first time a negative role of theanin on the inhibition of MRLC phosphorylation in HUVECs (Fig. 6, 7). On the other hand, our identified metabolites did not act directly as bioactive compounds when administered singly (Fig. 5). This information, along with the peak lists of unique concomitant factors serving as a metabolic fingerprint (Tables S1, S2, S4), will also be helpful for optimizing the use of green tea as a nutraceutical. We found several tens of metabolite peaks by OPLS-DA and PLS regression analysis; however, many remain unidentified. For reliable nutraceutical evaluation, it is necessary to extend the molecular coverage of identified metabolites in plants and foods. Together with further identification of metabolites, application of other ionization methods (atmospheric pressure chemical ionization, and matrix-assisted laser desorption/ionization), combinations of different separation modes (reverse phase chromatography and hydrophilic interaction chromatography), and/or the integration of other platforms (gas chromatography-MS and proton nuclear magnetic resonance spectroscopy) may be effective methods for enhancing the value and breadth of nutraceutical studies. At least, metabolomics-driven strategies such as the one described here may open new avenues in green tea-based functional food design. In nutraceutical and biomedical research fields, metabolomic techniques have been used to identify subtle metabolic differences between individuals, or between different factors or conditions, e.g., diet and formulation [1], [13]. Application of a metabolomic approach to evaluate the bioactivity of plants, plant-derived functional foods, or botanical multicomponent drugs may be useful for unraveling their complex mechanisms of action, and/or for exploring the complex interactions between foods/drugs and human health.

In summary, the aim of this study was to investigate the relationship between metabolomic data and bioactivity (health promotion effect) of diverse green tea cultivars. Our findings illustrate the usefulness of metabolic profiling with multivariate statistical analysis for evaluation of nutraceutical properties (in this case, the inhibitory effect on MRLC phosphorylation in HUVECs), for identification of bioactive green tea cultivars (Nou-6 and SR), and for identification of bioactive factors (polyphenols). Some bioactive factors that were enriched in SR were able to alter the function of extracts from YB, a major Japanese green tea cultivar, changing it from non-bioactive to active. Thus, the combination of a metabolomic approach and a bioassay, as a simple and effective methodology, may advance nutraceutical research. This has potential applications for the discovery of valuable plant cultivars and bioactive factors.

## Supporting Information

Figure S1 **Chemical structures of tea constituents.**
(TIF)

Figure S2 **Chemical structures of theanin showing the highest VIP value.**
(TIF)

Figure S3 **Chemical structures of 9 biofactors capable of transforming non-bioactive cultivars into bioactive ones.**
(TIF)

Table S1 **List of MS peaks focused by successive OPLS-DA.**
(XLS)

Table S2 **List of the representative MS peaks contributing to the separation among three representative cultivars.**
(XLS)

Table S3 **List of MS peak intensity of all variables in 43 cultivars.**
(XLS)

Table S4 **List of MS peaks of large VIP values (>1) in bioactivity-predictive regression analysis.**
(XLS)
