# Supplementary figures and images for: Metabolomics-Driven Nutraceutical Evaluation of Diverse Green Tea Cultivars
Source: PLoS One. 2011 Aug 10;6(8):e23426. doi: 10.1371/journal.pone.0023426 (PMC3154454; doi:10.1371/journal.pone.0023426)

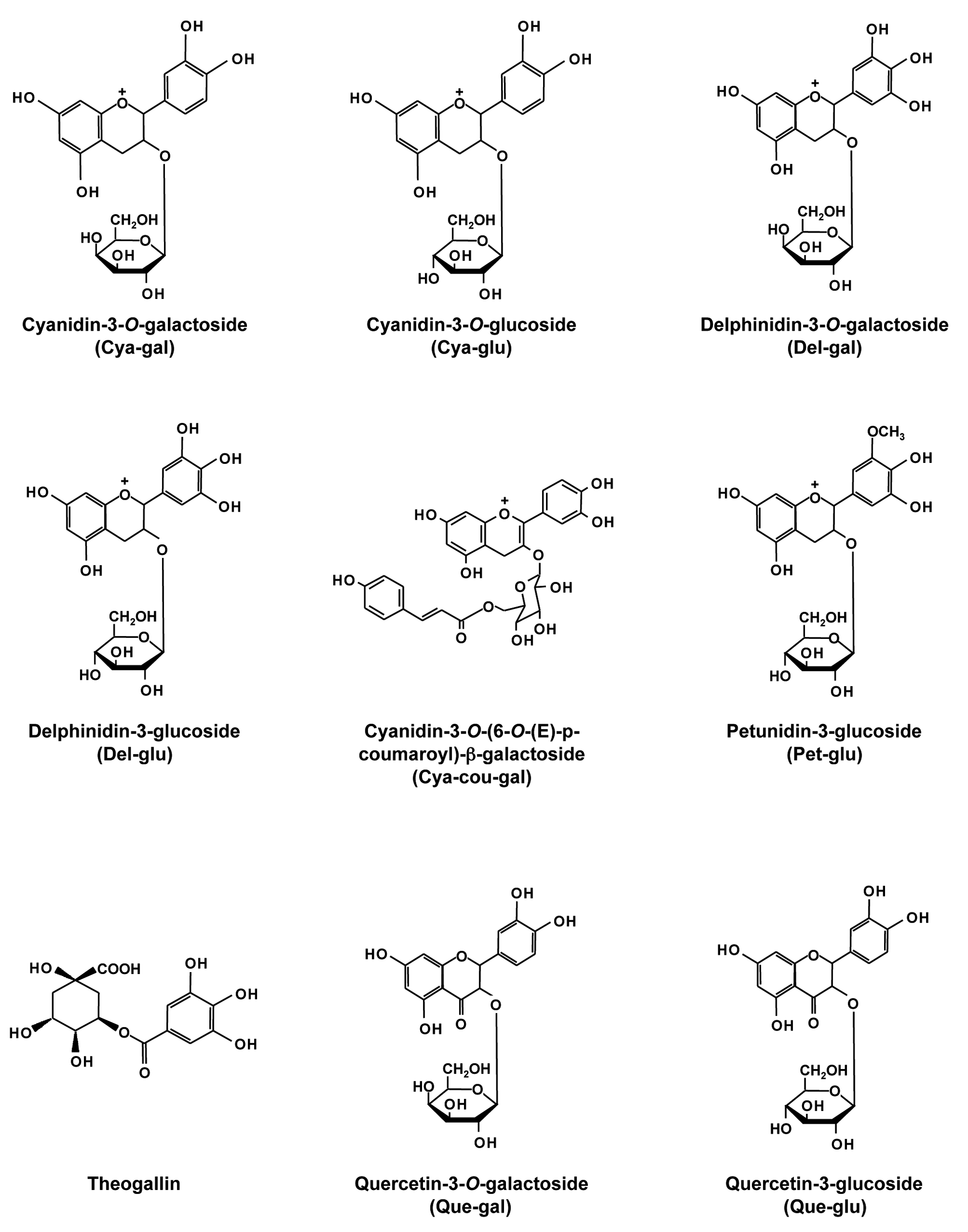

Supplement: Figure S1 — Chemical structures of tea constituents. (TIF) [file pone.0023426.s001.tif]

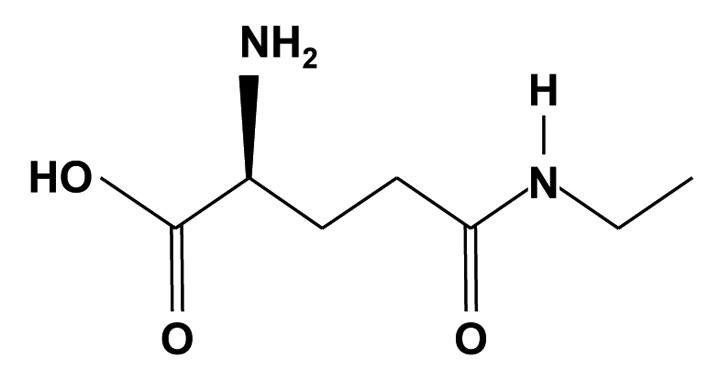

Supplement: Figure S2 — Chemical structures of theanin showing the highest VIP value. (TIF) [file pone.0023426.s002.tif]

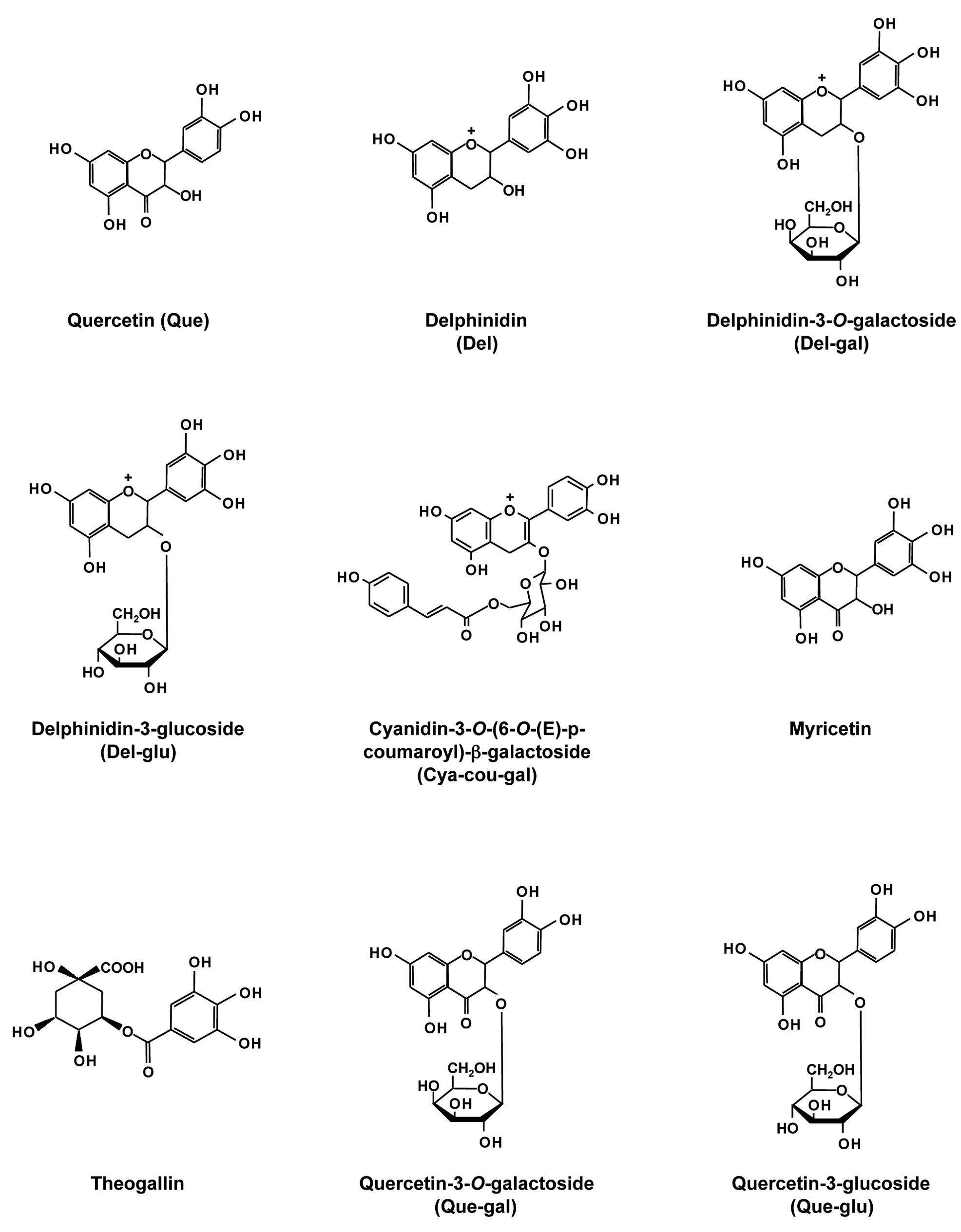

Supplement: Figure S3 — Chemical structures of 9 biofactors capable of transforming non-bioactive cultivars into bioactive ones. (TIF) [file pone.0023426.s003.tif]
